# *You Can’t Hold Their Hand the Whole Time*: A Qualitative Study of Parents’ Experiences of Adolescents With Food Allergy

**DOI:** 10.1111/jan.17002

**Published:** 2025-05-10

**Authors:** Meg O’ Sullivan, Rachel Flynn, James O’ Mahony, Juan Trujillo, Margaret Curtin

**Affiliations:** ^1^ University College Cork Cork Ireland; ^2^ Cork University, Hospital Cork Ireland

**Keywords:** adolescents, food allergy, paediatric, parents, qualitative, transition

## Abstract

**Aims:**

To explore parents’ experiences of parenting adolescents with food allergies.

**Design:**

An interpretive descriptive qualitative study.

**Methods:**

Semi‐structured interviews were conducted between November 2023 and March 2024 with 11 parents of adolescents with food allergies aged 12–16 years, 8 mothers and 3 fathers. Reflexive thematic analysis was conducted.

**Results:**

Three themes were generated: (1) Impact on everyday life, which concerns having to ‘always be on alert’, restriction as a family and mitigating feelings of difference in their child, (2) ‘handing over the reins,’ which describes parents role in the process of transition, including trusting their child, considering new adolescent socialising behaviours such as alcohol and intimate relationships, and ‘letting go' of some responsibility, and (3) learning, which details common misconceptions that parents still have and lessons learned from experiences of anaphylaxis and from tragedies in the media.

**Conclusion:**

This study adds substantial knowledge about the parental experience in food allergy, specific to parents of adolescents. Parents endure constant worry for their child, heightened at critical times, including the transition period of adolescence. Parents need guidance and support from healthcare professionals in this crucial time of change. Further education is needed as knowledge gaps remain even at this advanced stage in a parent's food allergy journey. Throughout the narrative, there was an undercurrent of parental anxiety, with periods of heightened anxiety illustrated in each theme.

**Implications for the Profession and Patient Care:**

The topic of transition and parents' role in the process should be introduced by healthcare professionals. Future work should focus on creating learning resources for families which cover the common areas of concern identified. Accessible education is needed for healthcare professionals without a background in allergy, particularly concerning anaphylaxis management.

**Reporting Method:**

The Reflexive Thematic Analysis Reporting Guidelines were used to guide reporting.

**Patient or Public Contribution:**

No Patient or Public Contribution.

**Summary**

- What does this paper contribute to the wider global clinical community?
  ○ Families should be advised to assess and mitigate risks, rather than trying in vain to avoid all risks through severe restrictions.
  ○ Clear, factual information should be made available to parents about common misconceptions, such as allergies being airborne, and situations where error is common, such as anaphylaxis management.
  ○ Guidance for parents on navigating emerging socialising behaviours of adolescents such as alcohol and intimate relationships is needed.

## 1 Introduction

Food allergy is a common condition, with studies suggesting a cumulative prevalence of 3%–6% (Sicherer 2011), with a strong impression that food allergy rates have increased over the past two to three decades (Sicherer and Sampson 2018). Food allergy most often begins in young childhood (Wood 2003), and therefore parents of adolescents have typically been dealing with food allergies for years. Consequently, parents have become practiced in how to manage allergies, predominantly avoiding allergens in all food eaten by their child and being prepared to treat allergic reactions, which can range from mild to anaphylaxis in the case of accidental exposure (Sicherer and Sampson 2018). However, this constant vigilance takes its toll, as it is well documented that parents or caregivers of children with food allergy experience an increased emotional burden (Golding et al. 2021). Despite this constant vigilance, due to the nature of food allergy, it is impossible to guarantee non‐exposure to allergens (Roberts et al. 2021). A study by Roberts et al. (2021) demonstrated a large proportion of parents of children with food allergy reported clinically significant worry, anxiety, and/or post‐traumatic stress symptoms, and that these were associated with intolerance of uncertainty. These poor psychological outcomes impact not only on the parents' quality of life, but also ultimately on their childs', as a feedback loop between parental and children's anxiety and coping skills is acknowledged (Bozen et al. 2020). Similarly, Chooniedass et al. (2020). found that parents wanted help with coping mentally with food allergies for both themselves and their child.

For an adolescent, many biopsychosocial changes occur at this age as they transition from dependent children to independent young adults. Correspondingly, in the parental–adolescent relationship, renegotiations regarding autonomy must also occur (Morris et al. 2021). In a study which examined the adolescent transition period in various chronic conditions, it was noted that parents found it difficult to relinquish responsibility for management of the condition and often parental anxiety surrounding the transition was more intense than that of the adolescent themselves (Coyne et al. 2019). In the context of food allergy, parents have less control over their teenagers' food allergy management as they are increasingly more independent, such as spending more time with peers rather than parents, contributing to heightened parental anxiety (Bozen et al. 2020).

Most previous research concerning parents of children with food allergies has broadly looked at parenting children of any age, as highlighted by a qualitative review (Moen et al. 2019). This review included 24 studies, with just three studies focusing on the adolescent period. All three looked at the families' perspectives, including both parents and adolescents, with one also including siblings. Overall, the review found two main syntheses; the families process of being confident with a food allergy and gaining knowledge about living with food allergy, a pathway in self‐education (Moen et al. 2019). Another relevant qualitative study was not included in this review as it was published the year after (Chooniedass et al. 2020). Although it includes parents of children with food allergies from 8 months to 15 years, the concept of transition emerged in the ‘ages and stages’ subtheme, with parents at the teenager stage requesting resources that cover ‘stepping into adulthood’ (Chooniedass et al. 2020).

There is a paucity of literature that exclusively focuses on parents of adolescents with food allergy, whose experiences and needs are likely to be different from parents of young children, and different from the young people themselves. Therefore, the aim of this study is to explore parents' experiences of adolescents with food allergies.

## 2 Methodology

### 2.1 Study Design

This study uses an interpretive descriptive design, as described by Thorne, which is a suitable methodology for addressing questions of lived experience, creating knowledge applicable to clinical nursing practice (Thorne 2016). Interpretive description fits within a constructivist paradigm, which acknowledges that an individuals' reality is built on a foundation of their context and experiences. Similarly, knowledge resulting from research processes is co‐constructed between participants and researchers, and thus the researchers' personhood and experience are a valuable asset, rather than a biasing limitation (Braun and Clarke 2022).

### 2.2 Participants and Context

Eligible participants were parents or guardians of adolescents aged 12–16 years who were diagnosed with an allergy to at least one type of food and prescribed an adrenaline auto‐injector (AAI). Recruitment was based on the adolescent being a patient of the paediatric allergy service of a large hospital in Ireland, which sees approximately 40% of children with allergies in the country (Gallagher et al. 2024). Upcoming clinic lists were examined and, if eligible, the contact parent or guardian was posted an invite letter, a consent‐to‐contact form, and a stamped addressed envelope. Potential participants who replied chose if they were to be contacted by post, phone, or email, and this was adhered to with all further contact. A total of 11 parents participated: 8 Mothers and 3 Fathers. Nine interviews were conducted, two of which included both parents at their request. The periods of dataset generation and analysis overlapped, as recommended (Braun and Clarke 2022). The concept of information power informed the decision of when to end the recruitment process, as congruent with reflexive thematic analysis (Braun and Clarke 2021). Information power indicates that the more relevant information the sample holds, the lower the number of participants is needed (Malterud et al. 2016). Five criteria support this decision: namely, study aim, specificity of the sample, established theory, quality of dialogue, and analysis strategy (Malterud et al. 2016).

### 2.3 Dataset Generation

The first author conducted the semi‐structured interviews, a female paediatric nurse and researcher who had no previous relationship with the participants. The interview guide was initially developed by the first author and then discussed and refined by the author group. Interview questions pertained to parents' experiences of their child's allergy in various settings now they are adolescents, how they as parents managed the transfer of responsibility to their child as they aged and support needs (see File S1). Questions were informed by literature, particularly guidelines on the effective transition of adolescents and young adults (Roberts et al. 2020) and content identified in a review of telehealth interventions for transition to self‐management in adolescents with allergic conditions (O' Sullivan et al. 2023).

Participants chose between either an online interview on Microsoft Teams or in‐person, in a university setting. Most participants selected online interviews, with just two conducted in‐person. The average interview duration was 45 min. Interviews were conducted between November 2023 and March 2024, at a time and date suited to the participants. Interviews were video‐recorded if the participant chose an online interview or audio recorded if they chose an in‐person interview. Interviews were transcribed verbatim by the first author, and for online interviews, the transcription feature of MS Teams was used as a base. Transcripts were then anonymised, with pseudonyms given.

### 2.4 Ethical Considerations

Ethical approval was obtained from the Clinical Research Ethics Committee of the Cork Teaching Hospitals (date of approval 29/09/2023, reference number ECM 4 (x) 12/09/2023 & ECM 3 (pp) 24/09/2023). Participation information leaflets were provided and potential participants were given the opportunity to clarify their understanding. Written consent was then given. No compensation was involved for participation.

### 2.5 Data Analysis

Reflexive thematic analysis was used to analyse the data by following six steps, as outlined by Braun and Clarke (2022). The first author became familiar with the dataset through repeated immersion, by conducting and transcribing the interviews and then re‐reading the data. NVivo software was used for initial coding, considering different possible interpretations and levels of meaning. The analysis then moved to traditional pen and paper techniques, as the first author found that physically writing and moving quotes around on a mind map board led to a more natural creation of patterns of meaning. An initial theme was discarded, with others refined, as authors remained focused on the study's aim. The flow of the overall story of parents' experience was central in naming and ordering themes. Deciding on which quotes best represented the central concept of each theme and contextualising the analysis in relation to existing literature concluded the writing up phase. Discussion among co‐authors was central throughout.

### 2.6 Enhancing Trustworthiness

Various techniques were utilised to ensure the overall trustworthiness of the study and that the four pillars of rigour in qualitative research are demonstrated (Lincoln and Guba 1986). Credibility is enhanced by debriefing among authors of varying expertise and perspectives. This includes a paediatric nurse with allergy experience and PhD student (MOS), a paediatric allergy consultant, researcher, and lecturer (JT), and lecturers in general (RF), paediatric (MC) and mental health (JOM) nursing with significant qualitative and other research experience. Reflexivity was also used to enhance credibility. Voice memos were made by the first author to capture observations in a quick manner at every stage. These were then incorporated into an online reflexivity journal, which was an opportunity to consider and explore beliefs and assumptions, and how they interact with the research process. A clear audit trail contributes to dependability, with many drafts of each step. Transferability is evidenced by a thick, rich description of the data and context. In addition, confirmability is established by the judicious use of participant quotes throughout the analysis to illustrate the clear links between the data and researcher interpretations. Further quotes can be found in File S2. The Reflexive Thematic Analysis Reporting Guidelines were used to guide reporting—see File S3 (Braun and Clarke 2024). These new guidelines allow for congruency with the values of Reflexive Thematic Analysis and advocate for reference to existing research and theory in the reporting of themes to add context to findings where relevant, rather than in the discussion alone.

## 3 The Analysis

Three distinct but interwoven themes were generated that illustrate the experience of parents. These described the impact on everyday family life of having a child with food allergies, the parental role of ‘handing over the reins’ in the process of transition, and the learning they have undergone to manage food allergy in their lives. Throughout the narrative, there was an undercurrent of parental anxiety with periods of heightened anxiety illustrated in each theme.

### 3.1 Impact on Everyday Life

Overall, parents described the pervasive nature of their child's food allergies, that impacted the family as a whole. The unpredictable threat of exposure of their child to their allergen contributes to the parental burden (Golding et al. 2021). Thus, anxiety and restriction are commonplace for parents, yet parents describe how they actively tried to impart a feeling of ‘normalcy’ on their child.

#### 3.1.1 Anxiety—‘Always on Alert’

Juggling the various aspects of food allergy management was depicted by parents as mentally draining. The constant risk of an allergic reaction in their child is frightening, with certain timepoints on their allergy journey being particularly distressing.

Universally, parents spoke of ‘*constantly worrying*’ due to their child's food allergy. They inferred that it is not the intensity of worry that is debilitating, but rather the consistency. The sense of unease is ever‐present, as ‘*it's just a risk all the time*’; no day goes by without eating. Parents were particularly anxious when their child was not with them, such as when out with friends, and therefore they couldn't control the situation. For some parents, their underlying degree of anxiety was particularly high. One parent, Mary, noticed a change in her own identity due to having a child with a food allergy; ‘*I don't think I'm the same person since Niamh was diagnosed*.’ Similarly, another parent noted how she feels her teenager moderates her own portrayal of fear because she knows her mother finds it difficult to cope with;

‘I think she hides it a bit from me as well. And she knows it scares me. Because it is scary.’ ‐Sarah

On top of the constant level of worry, parents collectively found certain periods particularly difficult over the years. One such time is waiting for an initial appointment after their childs' first allergic reaction. In Ireland, there is limited access to paediatric allergy specialists, with up to 48 months wait for non‐urgent assessment (Sheehan et al. 2024). One couple described the most ‘*horrendous*’ period being ‘*in limbo*’ waiting a ‘long, long time’ for their initial appointment with an allergy specialist, after their child's first reaction. They were discharged from the emergency department feeling unsupported, with just the advice of ‘don't eat nuts.’ Consultation with the allergy team gave them clarity as a family;

‘But then obviously you get tested and you realise what nuts she's allergic to. And then you're encouraged to introduce the other nuts and it actually helps.’ ‐Mike

Parental anxiety also peaked during times of diagnostic tests such as Oral Food Challenges (OFC). In an OFC, increasing doses of a suspected allergen are given to confirm a diagnosis of either an allergy or tolerance. The act of purposefully giving a suspected allergen to their child could be very stressful. As expressed by Linda, ‘*you can never have it, then to eat half a nut? We're not supposed to even look at these nuts*!’ However, while the test itself is often anxiety provoking, the results of the OFC, even if confirming an allergy, can reduce the parental burden, as indicated by the participants and in a systematic review (Kansen et al. 2018).

**FIGURE 1:**
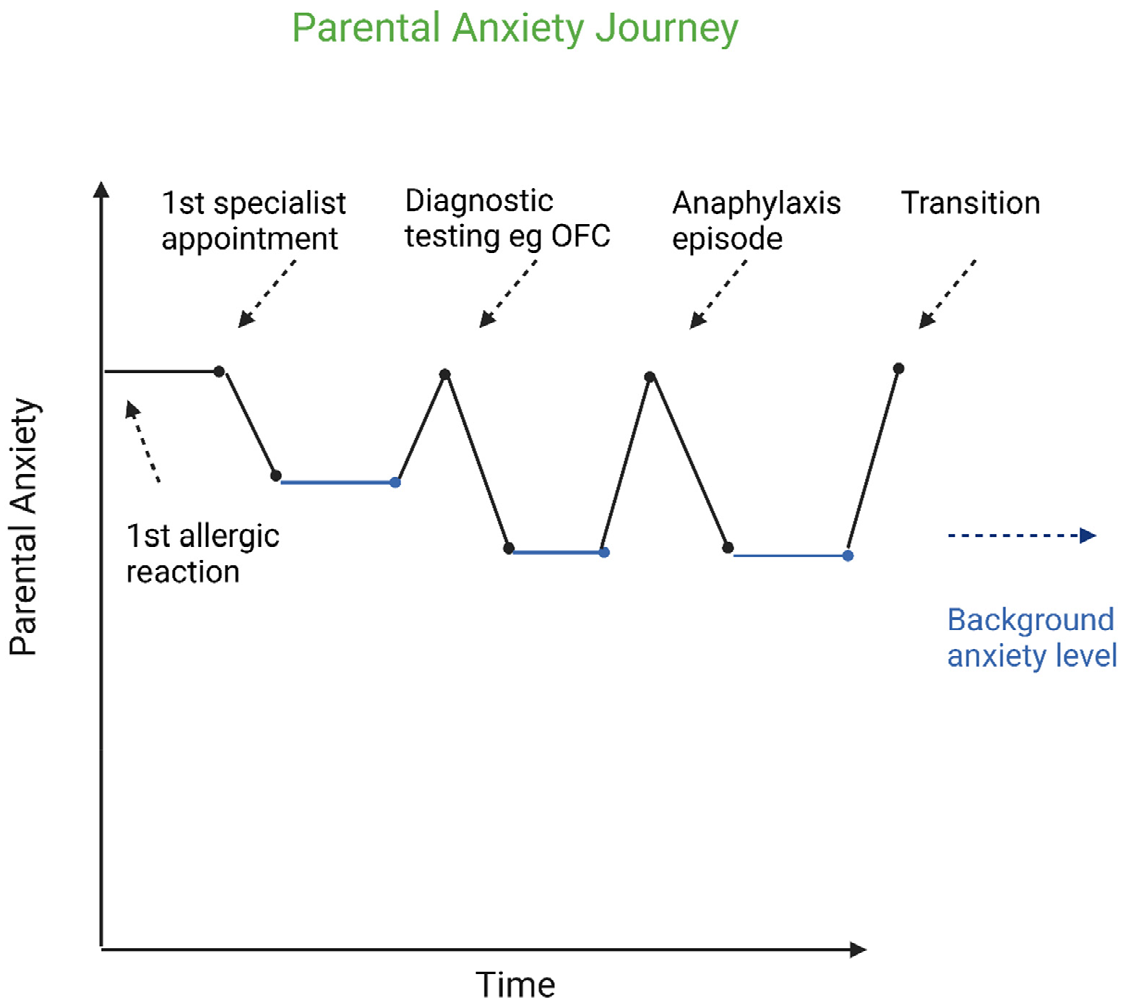
Parental Anxiety Journey. Events depicted in figure such as diagnostic testing and an anaphylaxis episode are to be viewed as examples and are not intended to mean that they always occur, and certainly not in this specific order.

This subtheme described how parents lived with a constant background level of anxiety due to their child's allergies, and how this can heighten at anticipated periods. This concept is illustrated in Figure 1, with further peaks of an anaphylactic episode and transition to self‐management for their child discussed in later themes. To manage this constant worry and feel safer, parents often apply restrictions to their daily lives, as explored in the next subtheme.

#### 3.1.2 Restriction as a Family

Many parents spoke of the limitations food allergy places on their day‐to‐day lives, particularly surrounding occasions. Following on from the previous subtheme, these restrictions were a natural result of anxiety about their child potentially having a reaction. They were also largely applied to themselves and other family members, including siblings.

Variety and difference in experiences were hindered, as some parents tended to avoid social activities, viewing them as not worth the risk. Going out for food was a particular issue due to the potential for allergic reactions and anxiety surrounding this risk. Participants found the unknown of somewhere new alarming, with most sticking to tried and tested food establishments.

And then if we find a restaurant and we get on well, we go back. A lot of holidays we barbeque. We like to, you know, be safe. We do eat out as well, but I do (pause). I won't lie, I do feel quite anxious. ‐Mary

Parents described making sure that the child with the allergy is catered for appropriately when deciding where to dine, with one parent stating that if the restaurant ‘*can't offer something then we'd all leave*.’ This sense of acting as together as a family was present throughout the data. However, parents were conscious of the effects of these restrictions imposed on siblings in the household with no allergy, and of possible resentment;

Cause my daughter said, ‘oh I'd love Chinese.’ And I says, ‘we can't risk it with Paul. ‐Linda

Restriction of parents' own diet were common, with some parents not keeping their child's allergen in the house at all. One parent describes the joy in having fish, her daughter's allergen, when her daughter was on a trip away;

It was only fish fingers, but just to have a few. I don't even like fish, but it was the idea, if that makes sense. ‐Sarah

Parents used restriction to manage their background anxiety level as they felt their child would be safer. Restriction as a family was used as a mechanism to avoid the child with an allergy standing out as different within the family unit, as explored in the next subtheme.

#### 3.1.3 Mitigating Difference—‘That Normalcy Bit’

Parents lamented the impact feeling different due to food allergies had on their child. In response, parents actively included them in activities with adjustments where necessary, both within the family and in the wider community. Where this was not possible, parents aimed to promote a pragmatic and resilient attitude in their adolescent child.

Unsurprisingly, parents highlighted that ‘*teenagers don't want to be different*’, and therefore disliked highlighting their food allergy. Instead of having to draw attention to his allergies, this parents' son tends to avoid the conversation altogether, reminiscent of the concept of ‘disclosure fatigue’ proposed by Schelly et al. (2023);

He'd be quite amm, embarrassed about it really. He'd more happily just sit there and not have anything, rather than say it. I've seen him at a friend's house and rather than say it to the parents, ‘I actually can't eat that because I've allergies', he just removed himself from the situation and went off. ‐Gillian

Parents felt possible ways of abating this uncomfortableness in their children of being different included them as parents treating it all as ‘their normal’;

‘And she hates her allergy. … But you're always looking for normalising the challenges. Because it is part of who you are and you're very normal.’ ‐Jessica

Parents spoke of wanting their child to deal with their worry and feelings of difference in a positive manner. Despite universally acknowledging being anxious themselves about their child's food allergy, several parents reported emphasising to their children that ‘*in the scheme of what's out there*’ other people's situations can be more difficult;

Like, you try, we try and play it down really, to not let it be a big deal.’ ‐Jo

Humour was used by some families in that ‘down‐playing’ manner. In addition to families' efforts, parents felt that teachers and friends understanding allergies help their children feel less like outsiders. Gillian recounted a year her son had a difficult time at school, which she felt was due to that teachers' poor understanding of allergies or lack of effort to include him in experiences;

Ok, well take for instance chocolate biscuit cake. They never informed me of it, so they just left him out. Whereas I was trying to explain to the teacher, if you inform me of these things, like, there's no reason why he can't be included. The only thing that needed to be substituted was the chocolate. I said, I could have provided the chocolate. He could have baked like everybody else.

This subtheme demonstrates how conscious parents were of their child feeling different, and the effort and thought that parents put into mitigating these feelings of difference as much as possible. Overall, this theme of impact on family life considered the constant anxiety parents faced which led them to restricting their families' activities, while wanting their children to deal with their anxieties and feelings of difference in a more positive manner.

This theme paid particular attention to timepoints of heightened anxiety, as illustrated by Figure 1. This includes the transition from them as parents being responsible for their childs' allergy management to their childs' self‐management, which is the topic of the next theme.

### 3.2 ‘Handing Over the Reins’

Parents recognised that as their child was now growing up, part of their role included preparing them for independent life. It is acknowledged that families can find it challenging to transition food allergy management from parent to adolescent (Herbert et al. 2022). This process involves parents building confidence in their child's ability to manage their allergies and trusting them, preparing for the new risks in adolescence, and finally stepping back into a role that supports their child to self‐manage rather than managing their allergy for them.

#### 3.2.1 ‘You Have to Trust Her Instinct a Little Bit More’

This subtheme describes how parents develop trust over time in their child's skill in managing their allergies and opportunities to further or impair that trust.

Almost universally, parents create a picture of the trustworthy, sensible child;

Like, we're very lucky that Conor would be a generally very responsible person anyway, which definitely helps with managing it. I think it's his personality. He's very trustworthy and that he'd know himself and wouldn't ever try and risk it. ‐Jo

This has parallels with a study in which entire families were interviewed, and they found that older siblings felt their siblings with peanut allergy were more mature, reflective, and perfectionist than themselves (Stensgaard et al. 2017). One parent recognised that she possibly relies too much on this maturity;

I do overestimate her on things. And I do forget she's still only 16… so I am guilty of doing that without realising I'm doing it. ‐Sarah

In order to participate in the process of transition, from the parents' perspective, they needed to have confidence that their child was competent in food allergy management. For example, ensuring their child consistently remembers to carry their AAIs is a major aspect of food allergy management that many parents reported currently trying to hand over to their child, while still supervising from a distance;

I used always be saying, ‘have you your [AAI]?’… Well, he's to remember about, you know, ‘cause I always had them. Now it's a case of he needs to bring them with him. ‐Linda

Parents noted that although their children may have struggled in the past with carrying their AAIs, this had substantially improved. However, parents were less confident in their adolescents' putting together of tasks into an overall competence in managing all aspects of food allergy, particularly a sense of intuition that parents have spent years building;

We have tended to look at the environment, assess it ourselves and then make a decision. But she needs to get that skill herself. ‐Mike

An opportunity for an adolescent to manage a situation without parental supervision had the effect of tipping the balance in the direction of doubt or confidence. As discussed by Herbert et al., ‘social experiences had the ability to both bolster and undermine feelings of safety and inclusion.’ (Herbert et al. 2022, 12) One parent described an incident where her daughter was out without her, in the company of friends. The adolescent was unprepared to navigate a restaurant situation independently, leaving her unhappy with her competence and contributing to feelings of difference;

Lately, she went somewhere now with her friend and her [friends] Dad spontaneously. And there was lots of texts. So she wasn't confident enough to tell the waiter herself. So I just told her to get a bowl of chips. But all her friends got dessert and she felt quite left out and a bit disillusioned when she came home. ‐Mary

On the contrary, some parents noted that their child having the opportunity to manage without their supervision, such as at camp or a school trip, had the potential to give them assurance of their own ability that they then carried forward. Parents are ‘*concerned about her moving out and going on*’ in the future, with finishing school looming in parents minds as an imagined deadline for which independence needs to be acquired;

Cause in another few years he will be going off to college, and going off himself. ‐Phil

This subtheme encompasses the development of trust in a parent that their child will be able to self‐manage independently and highlights aspects that parents find are going well and aspects that are more challenging. A particular wave of challenging aspects of food allergy management is specific to adolescence, and these are explored in the next subtheme.

#### 3.2.2 The Hidden Dangers of Adolescence

Parents repeatedly brought up typical adolescent behaviour that happens when teenagers are socialising without parents present, such as involving underage alcohol consumption and intimate relationships, and how they were particularly concerned about the potential consequences of these behaviours from a food allergy management point of view. They had a conflict between concern, while also not wanting to curtail them from doing ‘normal’ adolescent activities.

Following on from the previous subtheme, parents gained trust in their child's competence with allergy skills over time; with practice, supervision, and then independence. However, going out with friends and managing sexual relationships and alcohol was a major concern for most parents, contributed to by the fact that, unlike previous new experiences, they as parents would not be present to supervise. Many parents mentioned teenage discos as the first foray into this new world of independence. Managing the carrying of medication when socialising was a point of contention between parents and adolescents, particularly for boys, as girls were more likely to be wearing a handbag, as noted by some parents;

And like, say, if he was going to a disco, he's like, ‘I don't wanna be hanging, you know, having the pens [AAI] sticking out of my pocket.’ Which you're like, I get. ‐Jo

This reflects a study which showed rates of carrying AAI in those aged 13–21 years to be decreased for social activities such as dances (61%) or parties (64%), and when wearing tight clothing (53%) (Sampson et al. 2006). Kissing, which often goes together with discos, was another major concern for parents, such as this mother whose son has a milk allergy and is worried about him having a reaction;

But like, my biggest concern would be that if he goes to a disco and he, so he's at the age where he'll want to be kissing girls, like, how extreme is it, like if a girl was after drinking a cup of tea or eating a chocolate bar? Like, can you get an anaphylactic reaction from it? I don't know. These are worrying thoughts that I would have. ‐Gillian

Some parents have discussed and made their child aware of the possibilities of intimate relationships, telling them they could have a reaction ‘*if you start kissing a boy or whatever, and he's after having a packet of peanuts*.’ Some parents have relayed the information to their child, but in a light‐hearted manner;

We've made a few jokes about it. That it's like, oh, you better make sure she hasn't had a glass of milk before you kiss her. ‐Jo

Finally, their children drinking alcohol concerned parents due to their food allergies for several reasons, including potential allergens in drinks and becoming forgetful and mislaying their AAI. Parents also noted that ‘*with a couple of drinks, your inhibitions are dropped*’ and their adolescent could be less cautious of their surroundings, increasing the likelihood of an allergic reaction (Lee et al. 2024). Overall, parents worried about these hidden dangers of adolescence, and while some parents had some discussion about them with their child, many had not, as ‘*who wants to be talking about that with their mother*?’

These aspects of food allergy management in new situations were particularly concerning for parents, as their child would be experiencing them for the first time likely without their parents. However, parents recognised the developmental value of normal adolescent behaviour and letting them manage situations on their own, as explored in the next subtheme.

#### 3.2.3 Letting Go – ‘You've Got to Detach Yourself’

Parents acknowledged that their natural instinct was to strictly supervise their teenager and that having a child with allergies made them more likely to be ‘*helicopter parents*’ or overprotective (Westwell‐Roper et al. 2022). However, the need for letting go was well recognised as necessary for the wellbeing of all involved.

Universally, parents were aware of the need to transition food allergy management from themselves as parents to their child gradually, even if they were only at the beginning of that process;

I still make his lunches. So it's all, you know, it's all been, it's all safe as long as we do it… it's going to be more transferred to him as time goes on. ‐Jo

Nonetheless, this period of transition is yet another period of high anxiety for parents, as illustrated in Figure 1. Adolescence is a time of increasing independence and spending time with peers rather than family, and parents are anxious about the particular dangers of adolescent behaviour as discussed in the previous theme. This parental concern and anxiety surrounding extending safety zones and doing new things for the first time is recognised (Moen et al. 2019). Participants acknowledged that although their instinct is to keep them ‘*wrapped up in cotton wool*’ that this is not ultimately beneficial for their child's wellbeing. One parent described that in the past year, her daughter had several first‐time experiences, such going to discos, restaurants and the cinema without her parents, and ‘*Initially, I would sit outside in the car. Tap my fingers… We would just sit and wait, to be in the vicinity*’. However, they eventually began to trust her ability to manage and realised that they had to stop.

This concept of consciously taking a step back and detaching themselves as a parent was common throughout the data, with some suggesting that parents may need help with this;

So, I definitely think you do as a parent need a little bit of forcing out of it. You know, a little bit of back off, you've done your bit. It is now time for this child to take ownership, responsibility, independence. And the longer you sit on their shoulder the less likely they are going to do it and more likely they're going to get themselves into a situation that they can't manage because they've never had to manage, you know, and that's big learning for a parent. ‐Jessica

Similar to other stages on the allergy parent journey, the period of transition can be stressful and worrying for parents. However, parents realise that it is a necessary part of the journey to allow their child to ultimately be an independent adult, and thus it is a positive step;

The whole moving on to the next stage is inevitable. You'll be grateful that they can, I suppose. ‐Jo

This theme of ‘handing over the reins’ deals with the concept of transition from a parent's perspective, from preparing their child and learning to trust that they are now competent in food allergy management, managing the particular dangers of adolescence and a time of ‘firsts’, and finally to letting go of control and allowing their child to step up.

### 3.3 Learning

Part of the transition process involves parents imparting the knowledge they have gained over the years about food allergy management onto their children. However, if parents' knowledge of best practice and current allergy evidence is not reliable, then adolescents can unfortunately be the recipients of less‐than‐ideal information. This theme of learning explores common allergy misconceptions, how parents have learned from the experience of poorly managed allergic reactions and from prominent cases.

#### 3.3.1 Misconceptions

Navigating allergy myths and realities was a struggle for participants, despite them being parents to adolescents and having lived in the world of allergies for many years now.

Many common misconceptions were reiterated by parents. In some instances, these were causing parents more worry than necessary. One parent discussed trying to find a cause for her son's allergies, seeming to lay blame at her own feet.

So I felt, how could this happen, isn't breastfeeding meant to counteract all this? ‘Cause I really did. I breastfed the three of them. So I said how did this happen? Now I did, when I was pregnant, I possibly did eat chocolate peanuts. I did eat them, was that my fault? ‐Linda

Linda's narrative illustrates how misconceptions around allergy prevention can lead to self‐blame, impacting parental confidence.

Similarly, many parents mentioned ‘airborne’ food allergens, particularly on planes, seeming to be unaware that this is not a concerning potential cause of anaphylaxis. Most had grasped through experience that it isn't an issue for their child, with Linda realising, ‘now he doesn't seem to have a problem with it on your breath,’ and Jessica feeling relieved that ‘luckily she's not airborne that we know of.’ While these parents gradually ceased to worry about this supposed issue over time, they had spent years unnecessarily worried about it.

Regrettably, one parent reported being hyper‐cautious about this perceived risk, conscious of walking past a fishmongers, even after crossing the road, in case her fish‐allergic daughter could have a reaction from ‘*the fibres of my clothes*’ later in the day. Clearly, this belief about airborne allergens had caused this parent considerable worry and shaped her everyday actions.

In contrast, many parents illuminated misconceptions of theirs that had the opposite effect of making them not careful enough, through underestimating risk. Commonly, this was in regard to adrenaline autoinjectors. An example is believing that because their child has not had anaphylaxis before, then they do not have the potential to in the future, and so it is not necessary for them to be as strict with carrying AAIs at all times;

But I suppose we're probably more relaxed than we should be about making him bring them [AAI's], because he's never had to use them. ‐Jo

Similarly, another parent reported that if they did forget their AAI's when going out to eat, it's fine to just order something plain, contrary to the common advice of ‘no pens, no eating’;

‘And generally, if we go out for dinner, she'd always have her [AAI]. And if she didn't, if we were going out and she said, ‘oh my [AAIs], then she'd be extra cautious in the restaurant, you know, she'd have fish and chips.’ ‐Jane

This subtheme highlights common allergy myths, even among parents of adolescents. These misconceptions can affect parents actions, either in the direction of causing undue anxiety, caution, and self‐blame, or in the direction of partaking in risky behaviour unknowingly. Much of parents' knowledge comes from experience, which is explored in the next subtheme.

#### 3.3.2 Learning Through Experience

One of the most important aspects of food allergy management education is how to recognise and treat anaphylaxis. However, as anaphylaxis is thankfully not a regular occurrence, parents are often slow to identify the signs and symptoms, or are not comfortable with using an AAI. A standout feature from the data is how parents recounted in detail an incident in which their child was having an anaphylactic episode in which they did not give the AAI, but an allergy healthcare professional later told them that they should have administered it. Similarly, it was through the mishandling of an anaphylaxis episode that some parents learned the correct indications for treatment;

…my misunderstanding of when to use the pen [AAI] came into play, because when I spoke to [allergy staff], we should have used it because Niamh had a heaviness on her chest. And I thought she would have to be coughing and choking, which is not the case. So now I know. And I've told all the family. And I've told Niamh if she feels her chest is in trouble, we give the adrenaline. ‐Mary

In the course of these anaphylactic episodes, many parents brought their child to a local healthcare facility, and yet still did not receive adrenaline;

So I did go to [out of hours doctors facility] ‘cause I didn't know what to do, and he put her on the nebuliser, which [allergy staff] said he should have gave adrenaline. ‐Mary

This mother who is a nurse described bringing her child to her own place of work, and then to her General Practitioner's, where she received steroids rather than adrenaline and likewise, did not realise the error until she met the allergy team next;

I've been to [hospital] since and I told the nurse, or I told somebody that story, and they said you should have given her the [AAI]. ‐Jane

These parent experiences illustrate how a lack of parental knowledge directly influences the immediate health and safety of the child with food allergies. Parents discussed some other possible reasons why AAIs are not used in anaphylaxis, apart from not recognising which symptoms necessitated adrenaline. Other common concerns were fear of the injection itself and a reluctance to go to hospital. The mother who is a nurse acknowledged that despite her training, she failed to use the AAI when she should have and remarked that if it is an issue for her, it must be even more difficult for those without a background in healthcare, like most families with food allergies;

And I'd have some idea of what to do, whereas if you like, if you didn't have any, it's hard for a parent who's never given an injection to go straight in with an [AAI]. Do you know? And I've had lots of training. … And I still didn't know how to, when to give it! ‐Jane

Interestingly, some parents described that it was the calling an ambulance and going to hospital part of the allergy management plan that made them or their child reluctant to use an AAI, as that is what made it ‘real’ or a big deal;

I think what frightens Ava about an [AAI] administration is we've always had within her protocol… if you administer an [AAI], an ambulance has to be called. She has to go to hospital. And I think that's what prevents, that's her scare factor. I don't even think that it's the administration. I think it's the follow up. ‐Jessica

Similarly, this parent established calling an ambulance as a factor that dissuaded her from using the AAI;

Cause that night with the pen, I should have given it to him and I didn't. It was almost the last thing; I didn't want to have to do it. Call an ambulance and upset him. I was scared about it myself, that's the truth. ‐Linda

These anaphylaxis episodes were very stressful and were characterised by a sharp rise in parental anxiety, as denoted in Figure 1. These incidents also highlighted parents lack of knowledge and confidence in managing anaphylaxis.

#### 3.3.3 Learning From Shared Stories and Tragedies

In addition to learning through anaphylaxis experiences with their own children, parents also reported learning lessons from prominent cases in the media. Although death from anaphylaxis is rare (Umasunthar et al. 2013), there have been some high‐profile cases in recent years concerning adolescents, both in Ireland and around the world. Many parents brought up such cases, aware that there is an increased level of mortality from anaphylaxis in adolescence (Comberiati et al. 2019).

Parents also used these stories to discuss with their children the seriousness of potential reactions and the consequences of not following allergy management advice correctly, such as carrying AAI at all times;

And she's aware that the consequences are, that you know, it could be fatal… And I suppose we all know the story about the girl up in Dublin that time. So, she is aware of the seriousness of it. ‐Jane

One parent highlighted how, due to the death of an adolescent, lessons were learnt on a policy level and Irish law was changed to allow pharmacies to dispense adrenaline without prescription in an emergency (“Medicinal Products (Prescription and Control of Supply) (Amendment) (No. 2) Regulations” 2015);

Because actually, because of her, one time that Ava did get close to an anaphylactic reaction and we forgot the [AAI], I went to a chemist, the doughnut incident, as we call it. I went to the chemist and because of that poor girl, they had to give us [AAI]. ‐Jessica

This theme highlights how learning has occurred for parents, such as through their own experiences of managing anaphylaxis and debriefing with allergy healthcare professionals on what could have been done better, and through reiteration of the seriousness of the potential consequences of allergies and the importance of correct management through accounts of tragic cases. However, this theme also illuminates the dangers of misconceptions in food allergy, and the learning gaps these parents have, which need to be addressed in order to support their child's learning on their transition to independence.

## 4 Discussion

This study explores the experience of parenting an adolescent with food allergies via three interconnected themes, namely, the impact on everyday life, handing over the reins, and learning, with the undercurrent of parental anxiety woven among them.

Having a child with food allergies has a significant impact on parents' daily lives, with a lower quality of life consistently demonstrated (Golding et al. 2021). There can be a family‐wide impact of food allergy anxiety, which can result in limitations on activities (Westwell‐Roper et al. 2022). Restriction, particularly when it came to eating out, was a feature of the data in this study. Similarly, Crealey and Byrne (2023) reported that more than three‐quarters of Irish families with a child with food allergies only eat in food establishments that they have previously been to. Limitations such as these may affect relationships within the family unit, leading to feelings of resentment in siblings who do not have food allergies.

An Irish study showed that only 12% of adolescents aged 13–16 years with food allergy had been away from home alone (Crealey and Byrne 2023). Correspondingly, few parents in this study reported their child had been away without them, although of those who had, it was viewed positively. Thus, allowing opportunities for short, controlled periods of independence may build trust in a parent of their child's competence during adolescence, while they are still under their roof.

Typical patterns of teenagers socialising worried parents, as they identified allergy‐related risks. However, in contrast to eating in food establishments, parents in this study seemed conscious of not restricting their adolescent from having normal ‘going out’ socialising experiences. For example, most parents seemed to be allowing their adolescents to go to discos despite their concern, in contrast to a study which showed only 18% of adolescents with food allergy had been to a disco in the previous year (Crealey and Byrne 2023). This is encouraging, however parents do struggle with discussing these hidden dangers of socialising with their adolescents as they mutually find the topics uncomfortable.

Comparable to a study which examined transition through the lens of several chronic conditions, although parents understood the need for their adolescent or young adult to become independent, parents found it challenging to take a step back (Coyne et al. 2019). Parents need support and guidance from healthcare professionals to learn how to disengage from their active management role to a more supportive, nurturing role (Coyne et al. 2019). Ultimately, this permits a longer, more gradual transition period, benefitting adolescents (Roberts et al. 2020), whose best interests parents naturally wish to uphold.

Misconceptions about allergies are common, particularly about food allergies being airborne. However, a recent review by Turner and Dowdall (2024). demonstrated no evidence to support airborne transmission of nut allergens as a likely phenomenon. A study conducted in Ireland which examined food allergy beliefs found that 81% of mothers of children with a strong clinical suspicion of food allergy awaiting their first appointment with an allergy specialist thought that nut allergy was ‘airborne’ (Sheehan et al. 2024). Concerningly, such myths persisted despite the participants in this study being parents of adolescents who had been allergic for years. Misconceptions can be perpetuated by the media and anecdotal stories and can contribute to excessive anxiety, feeding into unnecessary restriction which further reduces quality of life.

Morbidity and mortality from anaphylaxis are associated with delay or lack of adrenaline use (Chooniedass et al. 2017). A UK study which surveyed allergic children and adolescents who had AAI prescribed found that of participants who had described a reaction in the previous year that met the criteria for anaphylaxis, 83% did not use their AAI (Noimark et al. 2012). The most common reasons cited for not using the AAI were that they thought adrenaline was unnecessary or were unsure if it was necessary. Other reasons included not having their AAI available or being too scared to use it (Noimark et al. 2012). Parents in our study also described reactions in which adrenaline should have been given but was not, with similar reasons given. In addition, parents reported avoiding using AAI as they assumed that the administration of adrenaline is why they are advised to call an ambulance after use. As Sicherer and Simons outline, it may be helpful for families to know they are seeking medical care not because of the use of the adrenaline, a safe treatment, but rather to assess and monitor the anaphylactic episode itself (Chooniedass et al. 2017).

In several of the anaphylactic episodes that participants recounted, they brought their child to a type of medical facility, and yet were treated with medications other than adrenaline. A French study which retrospectively examined allergy‐related visits to an ED found that of patients with a diagnosis of anaphylaxis at discharge, only 17% had received adrenaline; however, more than half received corticosteroids and/or antihistamines (Clark et al. 2023). While antihistamines, steroids, and inhaled β2‐adrenergic agonists such as salbutamol can have a role in anaphylaxis management, they are adjunctive treatments and are not appropriate for use as the initial or only treatment (Sicherer et al. 2017). As a result of these incidents, many parents in this study lacked confidence in non‐allergy health care professionals regarding allergy‐related care. A study by the European Academy of Allergy and Clinical Immunology (EAACI) working group on Primary Care showed that just 40.7% of European primary care health professionals surveyed had self‐perceived adequate confidence in food allergy care (Cabrera et al. 2022). As highlighted by Chooniedass et al., ‘there is a need for more non‐allergist health care providers to be educated on the topic of food allergies to meet the medical needs of their patients so that they are prepared and confident in their ability to manage a reaction’ (Chooniedass et al. 2017, 109).

Anxiety was a feature of all themes. This is unsurprising, as anxiety is known to be a pervasive state in the food allergy experience, as acknowledged by a two‐decade review of the literature surrounding the relationship between anxiety and food allergy (Polloni and Muraro 2020). Specific psychological support can be effective for highly anxious parents and families (Knibb et al. 2024), although the availability of these is unfortunately an unmet need currently (Knibb et al. 2019). A Europe‐wide survey of healthcare professionals in the field of allergy identified that just 25% of respondents had access to psychologists in their service (Khaleva et al. 2020). A practical goal for healthcare professionals is to promote a calm, matter‐of‐fact approach to parenting with food allergy, focusing on safety routines and adaptive coping, conveying that food allergy is manageable (Polloni and Muraro 2020).

## 5 Strength and Limitations of the Work

A strength of this research was the methodological congruency with which it was conducted and the variety in expertise of the authorship team. A limitation is that participants were recruited from one centre, although this does cover a wide geographical region, and so the analysis is likely to be relevant and transferrable to other contexts. Demographic information such as average age, race and ethnicity, and education level was not collected from participants. This is a limitation, as it could potentially have added further context to the analysis. The majority of participants were mothers, as is common with research concerning parents. As a result, it is possible that findings resonate less with fathers, as previous research has suggested that mothers feel more empowered but experience lower food allergy‐related quality‐of‐life compared to fathers (Warren et al. 2015).

## 6 Implications for Policy, Practice and Further Research

Families should be advised to assess and mitigate for risk, rather than trying in vain to avoid all risk through severe restrictions. Clear, factual information should be made available to parents about common misconceptions and situations where error is common, such as anaphylaxis management. Guidance for parents on navigating emerging socialising behaviours of adolescents, such as alcohol and intimate relationships, is needed. In addition, healthcare professionals should ensure these areas are addressed with adolescents directly, as many families do not discuss them due to discomfort. The topic of transition and parents' role in the process should be introduced by healthcare professionals. Brief, organised periods of time in which an adolescent is away from parents should be encouraged to build confidence ahead of more permanent independence, such as moving out of home.

Future work should focus on creating learning resources for families, which cover these common areas of concern. This would support the smooth transition of responsibility from parents to adolescents, as trusting their child's allergy management capabilities is essential for parents to feel secure in stepping back. Improving access to clear and concise educational materials can also have the beneficial impact of reducing anxiety (Polloni and Muraro 2020), particularly at key points identified in Figure 1. There also needs to be a mental health component to this education so that caregivers understand that symptoms such as anxiety and fear are common and can be managed (Chooniedass et al. 2018).

Succinct, accessible education is needed for healthcare professionals without a background in allergy, particularly concerning anaphylaxis management.

## 7 Conclusion

This study adds substantial knowledge about the parental experience in food allergy, specific to parents of adolescents. Parents endure constant worry for their child, heightened at critical times, including the transition period of adolescence. Parents need guidance and support from healthcare professionals in this crucial time of change. Further education is needed as common knowledge gaps remain even at this advanced stage in a parent's food allergy journey. These insights into parents experiences may help healthcare professionals understand better parents actions and behaviours in this stage, and thus be better equipped to care for the whole family.

## Author Contributions

Meg O' Sullivan, Rachel Flynn, James O' Mahony, Juan Trujillo and Margaret Curtin made substantial contributions to conception and design, or acquisition of data, or analysis and interpretation of data. Meg O' Sullivan, Rachel Flynn, James O' Mahony, Juan Trujillo and Margaret Curtin involved in drafting the manuscript or revising it critically for important intellectual content. Meg O' Sullivan, Rachel Flynn, James O' Mahony, Juan Trujillo and Margaret Curtin given the final approval of the version to be published. Each author should have participated sufficiently in the work to take public responsibility for appropriate portions of the content. Meg O' Sullivan, Rachel Flynn, James O' Mahony, Juan Trujillo and Margaret Curtin agreed to be accountable for all aspects of the work in ensuring that questions related to the accuracy or integrity of any part of the work are appropriately investigated and resolved.

## Conflicts of Interest

The authors declare no conflicts of interest.

## Supporting information


**File S1.** Semi‐structured interview schedule.

**File S2.** Additional quotes.

**File S3.** Reflexive Thematic Analysis Reporting Guidelines (RTARG).

## Data Availability

The data that support the findings of this study are available from the corresponding author upon reasonable request.
