# Supplementary material for: You Can’t Hold Their Hand the Whole Time: A Qualitative Study of Parents’ Experiences of Adolescents With Food Allergy
Source: J Adv Nurs. 2025 May 10;82(1):732–44. doi: 10.1111/jan.17002 (PMC12721920; doi:10.1111/jan.17002)
Supplement: Supplementary file 1 — File S1. Semi‐structured interview schedule. [file JAN-82-732-s003.docx]

**Supplementary file 1: Semi-structured interview schedule**

1. Tell me about your experience of managing your child’s food allergies now that they are a teenager?
2. At home
3. When they are not with you
4. Have you had any issues in transferring responsibility for allergy management to them as they are getting older? Are you worried about this for the future?
5. Do you feel your child needs more support from your healthcare team? In what aspects?
6. Education – like understanding food allergies and how adrenaline works,
7. Training - skills like how and when to use their adrenaline auto-injector, how to communicate their allergy to staff when eating out,
8. Monitoring – food labels and keeping track of any reactions,
9. Emotional support - coping with allergies and issues like stress or bullying, communication with friends and family,
10. Transition support - preparing to move to adult centred healthcare, understanding financial resources,
11. Any other aspects.
12. How best could we deliver that support do you think, bearing in mind limitations of the service and your families own schedule?
13. Would you be open to what we call ‘telehealth’, which is remote healthcare using for example videoconferencing or a mobile application?
14. Would you prefer support to be individual or in groups?
15. Would you prefer support to be one or several sessions?
16. Do you think parents should be involved in the intervention also or just the adolescents themselves? If you think parents should be involved, in what way?
17. Would your child use such a support if it was available?

*Footnote: data on preferences for self-management support is not analysed in this paper.
