# Supplementary material for: You Can’t Hold Their Hand the Whole Time: A Qualitative Study of Parents’ Experiences of Adolescents With Food Allergy
Source: J Adv Nurs. 2025 May 10;82(1):732–44. doi: 10.1111/jan.17002 (PMC12721920; doi:10.1111/jan.17002)
Supplement: Supplementary file 2 — File S2. Additional quotes. [file JAN-82-732-s001.docx]

**Supplementary file 2 – Additional quotes**

Caption: This supplementary file contains examples of some additional quotes from participants that could not be included in the main text of the paper, and full quotes where quotes have been shortened in the main text of the paper.

**Theme 1: Impact on everyday life**

**a: Anxiety - ‘always on alert’**

‘*That’s the big thing, having to think about it all the time’*

- Phil

*‘Like, you’re gonna always be conscious of it.’*

- Jo

*‘But am, you know, you are constantly asking, asking.’*

- Pat

*‘You know it’s, it’s just a risk all the time, I think… I don’t think I’m the same person since Niamh was diagnosed. Definitely not. I’m always on alert. Always. Yeah.’*

- Mary

*‘Its just the (pause), the inconvenience of remembering all the time.’*

- Linda

*‘Ah you would be constantly worrying, constantly worried that something might happen to him when he’s out of your care like. But you just have to learn to, (pause), yeah.*

- Phil

*‘Every time he’s out at a sleepover or something, I’m going, ‘Oh God’, you know, I’m just getting nervous.’*

- Linda

*‘I suppose it’s kind of scary enough at times, to be honest with you.’*

- Pat

*‘He is a bit of a worrier...I remember he used to be at the childminder and there was this other girl there and like he’d ask, ‘is there nuts in that?’ And he might ask the exact same question the next day, and she goes, ‘for God’s sake, he asked that yesterday!’ But that’s just the way he was like, he was constantly asking.’*

- Phil

*Jessica: ‘I suppose that challenge on that day did two things really*

*Mike: Scared the crap out of us? (laughs)*

*Jessica: Reiterated that yes, she’s absolutely got a freaking nut allergy. This is like, there’s no question about it. But it also, I suppose, reiterated the fact that she didn’t react hugely to small quantities… so I think that might have given us some confidence.’*

*‘She’s very afraid of it. I think that’s the bottom line. She’s very afraid of it… And she hates her allergy. She hates it. Because she does understand the severity.’*

- *Jessica*

1. **Restriction as a family**

*‘Like is it worth even doing this or going there, if something happened to him.’*

- Linda

*‘But you know what, you’re always conscious of it. You know, if you’re going out for dinner, we’ll only go to somewhere where we know he’ll get to eat anyway, and that it’s trustworthy. Because you will have some places that will guarantee there’s no dairy and there’s no eggs, and we've been caught with it before. So we kind of know where we’ll go and not go, but we kind of do it as a unit, as a family, really. If he, if Conor is not going to get something he’d like to eat, then we won’t bother going there… And we’ve actually, where there’s nothing, that they can’t offer something then we’d all leave, kind of go, look, sorry, it’s just that, you know, he can’t eat’*

- *Jo*

*‘We try to cook from scratch as much as we can…. Yeah, we’d be careful where we go’*

- Sarah

*‘She doesn’t adventure too much. She did have a Japanese now the other day. And she said, ‘oh God, can I, you know, is there anything?’*

- Jane

Mike: *They refused to serve us a sandwich, actually. [chain restaurant name]. Yeah.*

Jessica: *We got refused. They wouldn’t feed her. And all I wanted was a cheese sandwich, but because I said she had a nut allergy they wouldn’t give her anything to eat. No, it was horrendous.*

Mike: *And that was early days, but not really that early. They wouldn’t even turn the board over, wipe it down. They would do nothing.*

Jessica: *‘We don’t deal with that here’. That’s exactly what they said. ‘We don’t deal with that here’.*

*‘And then like, when Niamh goes to parties now, I don’t go with her, but I do get a list of ingredients and we make choices before she goes. She wouldn’t ever have birthday cake.’*

- Mary

*‘A lot of times we just takeaway and eat at home. We’re always scared of it to be honest.’*

- Linda

1. **Mitigating difference– ‘that normalcy bit’**

*‘And to just make it normal. If other people in school are going, ‘oh what’s that?’ If other people knew about it, it mightn’t be such an issue for people then.’*

- Linda

*‘So we’ve kind of stopped the aeroplane announcement thing. She never liked it. She hates that difference. She hates it. Even if I said to her, look, nobody on this aeroplane knows it’s you. Not a sinner knows it’s you. You’re fine, work away. She did not like it.’*

- Jessica

*‘I mean, emotionally, she’s very accepting of what she has… in the scheme of what’s out there, it’s a small thing for her.’*

- Jane

*‘And so I suppose there’s obviously pro’s and con’s, but we kind of strive to not, you know, say if someone does make, uh, you know, like say, yeah, I’m trying to think, there was some comments before and we were like, no. Like, you try, we try and play it down really, to not let it be a big deal.’*

- Jo

*And there have been times that she has turned around and it’s been particularly in that difference thing where she has said ‘I really wish I didn’t have this allergy,’*

- Jessica

*‘But she kind of half laughs it off. Not that she finds it funny, but I think it’s probably better to kind of have some comedy with it, or, you know what I mean… We’ve a joke, like I call her Angelina Jolie (laughs). Again, just to kind of, you know what I mean? Or if you’re going on a date, we’ll get some fish [her daughters allergen] for your lips. But just a joke like. Anything to just kind of (pause). Yeah, things like that. So yeah, that’s how we cope with it.’*

- Sarah

*‘I think for her going forward, you do kind of feel like you’re always on a knife’s edge kind of thing, you know?... So I do kind of feel, as an adult, and as, and for her going forward, there is that element of uncertainty, you know? All the time. But I suppose it’s how you manage that. But that’s that emotional resilience bit.’*

- Jessica

**Theme 2: ‘Handing over the reins’**

1. **‘You have to trust her instinct a little bit more’**

*‘I would say she’s very switched on. She’s very clued in.’*

- Jessica

*She’s very organised and in control of everything to do with herself.’*

- Jane

*‘Even though, out of the two of us, she’s the bright one. She’s the one that knows all the bits and bobs. But at the same time, I’m the safety net… I do overestimate her on things. And I do forget she’s still only 16… so I am guilty of doing that without realising I’m doing it because she’s always been… This is so like, sometimes I think if I ever had another child, which I won’t, but how would I cope if they were different. Because Emma’s on the ball, you know what I mean?*

- Sarah

*‘And if there’s treats like [chocolate] and crisps, you know, she knows what to have and what not to have. And she’d be very good even if there’s a bowl of food that others have handled, she won’t put her hands in, you know.’*

- Mary

*‘He’d never eat anything without double checking.’*

- Phil

*‘I know it sounds a bit mad, but they’ve gone from 60% compliant bringing them [AAI] everywhere, to probably 98% compliant bringing them everywhere.’*

- Pat

‘*She would, 99.9% of the time, opt out of eating something that she’s not sure of… I’ve just only seen her once take a chance. And actually, because it happened so infrequently, that one time she did take a chance, I nearly fainted. I was like, are you actually serious? You ate that chocolate bar not knowing what was in it… I couldn’t believe it… I said but you didn’t know, it could have had the brazil nuts, it could have had the walnuts. So I’ve only ever seen her do that once.’*

- *Jessica*

Gillian: *‘He’s very good if we’re not around. He, he knows how to read packets, he knows himself what he’s allergic to. He’s very aware of his allergies.’*

Christopher: *‘But if he’s going to a match, he could happily go out and not bring the bag.*

Gillian: *‘Yeah, it’s getting him to remember his medicine bag is the issue constantly.’*

*‘So he had none of those incidents [allergic reactions when at camp], which was brilliant and built, you know, his confidence and all our confidence of when he’s away as well.’*

- Jo

1. **The hidden dangers of adolescence**

*‘It’s the socialising and stuff with getting older. So to me, to me right now that is, that is the huge, the biggest one [worry].’*

- Pat

Mary: *‘Yeah, but I do think it’s going to get more difficult out in the big bad world with, you know, teenagers.’*

Interviewer: *‘And is there anything in particular that you’re maybe a bit more worried about for the future?*

Mary: *‘Well, probably kissing boys! If they’ve eaten peanuts or nuts, yeah. Yeah, and alcohol. Alcohol as well, with alcohol you don’t know what’s in it.’*

*‘Of course, you know, when you’re with your friends you’re going to be doing stuff, and whatever… as all of us probably when we were young.’*

- Jo

*‘It’s, you know, it is a matter of life and death at the end of it. If they do get it wrong.’*

- Jane

Interviewer: *‘is there certain things you can think of that they’ve started to do themselves?’*

Pat: *‘There is. I mean, lets, I mean the most obvious one is those fucking teenage discos! (laughs).*

‘*As Roberts getting older now going out and stuff, just even like, I’d love to meet other parents or hear from other parents on how, especially boys, how they carry their [AAIs] and [antihistamine] and stuff going out. Because girls have their bags and stuff, but the boys, I dunno. I just find it hard to (pause). Kind of worrying as to how he’s going to bring it.’*

- Gillian

*‘Yeah, I suppose it is dropping your (pause). It’s like all of us, with a couple of drinks, your inhibitions are dropped. You know, your awareness.’*

- Pat

*‘If they’re having alcohol, you know you’re, you’re less aware of, you know, if he didn’t have any alcohol, you know, he’s not going to put himself in that situation.’*

- Jo

*‘And it’s only recently I thought about the drink side of things. My other son is 16 and he said to me, ‘this time next year I’ll be going out.’ And I was thinking, right, how are we going to cope with Paul who has a nut allergy going out with two [AAI], he’ll probably lose them.’*

- Linda

*‘You know when you have a drink there’s always peanuts around, isn’t there. That’s a worry.’*

- Linda

*‘Stella [Their Mother] does a lot of reading up and stuff, you know, and there’s actually, you know, there’s actually different brands of alcohol, or different versions of alcohol where actually there’s nut involvement in it. Ah, you know, types of certain vodkas and, or maybe vodka in general, but Stella has done it. And some of the gins and what have you. And you know, while we haven’t gone fully, fully into it, it was something we weren’t aware of.’*

- Pat

1. **Letting go – ‘you’ve got to detach yourself’**

*‘Like, you’ve got to allow for that move to happen and you’ve got to detach yourself. And I think as parents of kids with allergies, you’re more likely to be that helicopter parent, so it’s harder for you to let go and to allow for that child to become the developing independent young adult that they should be.’*

- Jessica

*‘And I think we've done all the, it's not work now, it’s not work, but we've done all the donkey work up to this point and we've looked after them up to this point… You know, it all goes back to the wrapping up in cotton wool. There comes a time you have to say, look, you can’t keep, you know, you can’t lock them away all, all their lives’*

- *Pat*

*‘And as much as I would love to, as her mother, sit on her shoulder for the whole of her life, and manage and control and support and mind and coddle, and do all the things that you shouldn’t do. You can’t, you know, and I mean, we’ve had learning because in her fifteenth year of life, we’ve had to allow her to engage in experiences that have put her out of our sight. And we’ve had to make ourselves do it. Like there’s, in the initial stages, I would say it’s like, remember the first disco that she went to, the first restaurants that she went to, all like, 12 months, within this 12-month period. Initially, I would sit outside in the car. Tap my fingers. We went to the cinema one time. We would just sit and wait, to be in the vicinity. But we’ve had to, for her sake and for our sake, we’ve had to allow. No, you go. You do.*

- Jessica

*‘So I suppose in one way, up to this point, we’ve done it and we’re delighted, amm, that we managed to keep them safe. But I suppose there is just that thought process now coming into the back of minds. You can’t hold their hand the whole time, you know.*

- Pat

**Theme 3: Learning**

1. **Misconceptions**

*‘Like where she does drama on a Saturday, there’s a resource centre on [street] and there’s a fishmonger’s outside, so we, and it’s a wide street, and we try and avoid it when we can, but we cross the road, and cross it again. And afterwards, when I’m walking back after Emma’s gone it, I always have it in my brain, like sugar, should I be walking past them?... Like, will it get onto the fibres of my clothes when I see her at 5 o clock? Can she take it up from me?*

- Sarah

1. **Learning through experience**

*‘I had [AAI] all my life, I gave him [antihistamine]. I should have given the [AAI], I believe since. Gave him [antihistamine] and he got sick, vomiting, vomiting. Then I realised it’s coming out at least. So we never did give the [AAI]. But I had an appointment the following day, my yearly appointment…And I told them, they said, ‘oh, he should have had the [AAI] straight away.’*

- Linda

*‘…and about an hour and 40 minutes in, Robert started getting really caught for breath, the continuous cough and runny nose. So we just put him in the car and we have given him [antihistamine] and his inhalers and put him in the car and started bringing him to hospital and on the way we pulled in the car and we were just about to administer it, but he started to come around. But they actually said to us we should have administered it, straight away, yeah.’*

- Gillian

Gillian: *Yeah, I suppose all along I kind of was naïve to really, I suppose, I don’t know, like, I thought like, the major reactions that he would have had, because he had this since six months, was the coming out in hives or the blisters, the swelling of the lips, things like that. But I found for that that reaction that he had that day, there was none of that. Only a constant cough. And that’s something that I actually didn’t know about, or the runny nose. And they say when he’s in distress, those can be the first symptoms. I suppose I didn’t really know that at the time.*

Christopher: *And the delay, the delayed reaction.*

Gillian: Y*eah, that we didn’t, we thought that if he was to have a reaction, it was going to happen straight away. We didn’t realise that there could be up to two hours of a delay.*

*‘So she was at her friends house, I’d say around two years ago, and she ate a little bit of a protein ball and knew instantly herself that there was brazil nuts in it. And the Mom contacted me and she was okay. So I told her to give antihistamines and I’d call up. I was working, but I was actually at home, and I brought her [AAI] pens from home to the house. And by the time I got to the Moms, to Ellen, she was having a full-blown anaphylactic reaction. She was like a lobster. She was itching all over. Now her throat wasn’t swollen, but she was tearful. She had everything else. So I brough her straight to the [hospital] where my office was and I rang the director there. The CMM 3 was there and he came up. I said bring adrenaline with you. And anyway, I rang the GP and the GP told me give her hydrocortisone. And I said, well how much of that do I give her? So he said 30 milligrams of the prednisolone. So I gave her the 30 milligrams, and I brought her down to the GP surgery. And the CNM 3 came with me with the adrenaline, because it was COVID times. So we waited an hour outside the GP and she was fine. I’ve been to [hospital] since and I told the nurse, or I told somebody that story, and they said you should have given her the [AAI].*

- Jane

*‘…so I put the almond butter on his crackers. And within a couple of minutes his sister was saying, ‘is Paul okay? He’s getting awful red,’ she said. As I was looking at him, he was changing. His eyes were watering, his face was swelling, his mouth was getting (pause). And I looked at the stuff I had given him, peanut and almond spread, mixed. I took the wrong thing out of the press. So I went down, my husband was down the garage. I said, ‘what will we do, I think I’ve given him peanut.’ So he came up and went, ‘oh my God’. So we gave him (pause). I had [AAI] all my life, I gave him [antihistamine]. I should have given the [AAI], I believe since. Gave him [antihistamine] and he got sick, vomiting, vomiting. Then I realised it’s coming out at least. So we never did give the [AAI].*

- Linda

Mike: *I think that she didn’t have a great, we’re here next to a local hospital, which is only an injury clinic.*

Jessica: *Horrendous.*

Mike: *And they didn’t know what to do. They didn’t administer adrenaline. It was only when the paramedics arrived.*

Jessica: *Yeah. And I think that frightened the shite out of her.*

Mike: *They were looking at her like, what’s going on?*

Jessica: *They gave her [antihistamine]. They gave her [antihistamine]. They got the crash cart out. And at this stage we didn’t know what*

Mike: *Her eyes were swollen.*

Jessica: *We kind of figured it was an allergy…. But when we got to hospital, their answer was to give her [antihistamine]. They fought with the paramedic. The doctor got the crash cart out, was ready to start (pause), whatever else he was going to fucking do with the crash cart, excuse my French. But it was a paramedic that came down and looked at the doctor and said, ‘have you administered epinephrine?’ Isn’t that what he called it? I can see the conversation and I can see the paramedic in front of me. He was amazing. And the doctor said, ‘oh no, no, no, she doesn’t need it, we’ve giver her [antihistamine].’ And the paramedic said, ‘no, I’m going to take over from here. Excuse me.’ And he literally pulled the doctor out of the way, and I’m nearly sure he took it out of his sleeve.*

Mike: *Yeah, he had it on him.*

Jessica:  *I’m nearly sure, I know the bag was there, but I’m nearly sure he took it out of his sleeve. And he immediately administered the epinephrine.*

Mike: *It was almost instant.*

Jessica: *And instantly she started to be okay. And then they brought her across to [larger hospital], which they were super in. I mean, it was a different scenario, she had come out of the allergy. But I think that, probably that experience was enough to kind of put the fear of God, like oh God, that was horrendous. It was horrible. I don’t know how aware she was of it though. She has heard us talking about it, and I’ve certainly always said, ‘if I ever get into an emergency ‘tis a paramedic I’m going to be looking for, not a doctor.’ So she’s aware of it, but I don’t know how much she remembers, between an age thing, and she was very distressed and anxious. And she was, she wasn’t losing consciousness, but she was drowsy, she was struggling.*

Mike: *Like, you could see she was struggling. Her saturations, her blood, blood oxygen levels were probably low, yeah, she was definitely not great.’*

1. **Learning from shared stories and tragedies**

*‘Am, but now that we’ve got to this stage and they were 15 there last month and all that... And I suppose that’s where the, because unfortunately, that’s where the stories are in the media over the last couple of years, as you know yourself. And the girls, you know, are more aware of it aswell because they’re reading these stories.’*

- Pat

*‘I remember that girl died in Dublin a few years ago and that kind of brought it up. It’s just very sad. I think it made all of us more aware.’*

- Linda
