# Supplementary material for: You Can’t Hold Their Hand the Whole Time: A Qualitative Study of Parents’ Experiences of Adolescents With Food Allergy
Source: J Adv Nurs. 2025 May 10;82(1):732–44. doi: 10.1111/jan.17002 (PMC12721920; doi:10.1111/jan.17002)
Supplement: Supplementary file 3 — File S3. Reflexive Thematic Analysis Reporting Guidelines (RTARG). [file JAN-82-732-s002.docx]

# **Supplementary file 3: Reflexive Thematic Analysis Reporting Guidelines (RTARG)**

# Caption: These new reporting guidelines were created by Virginia Braun & Victoria Clarke to be coherent with the values of reflexive thematic analysis. This features in the reference:

Braun V, Clarke V. Supporting best practice in reflexive thematic analysis reporting in Palliative Medicine: A review of published research and introduction to the Reflexive Thematic Analysis Reporting Guidelines (RTARG). *Palliat Med*. Published online June 1, 2024. doi:10.1177/02692163241234800

| **Advice for aspects of the research report/approach to reporting** | **Guiding notes and further explanation** | **Practices, concepts and terminology to avoid** | **Section in**  **Main text file** |  |  |
| --- | --- | --- | --- | --- | --- |
| **The Introduction**  NB: We prefer *Introduction* over *Literature Review* as a section heading, to capture the broader purpose of this section. | | |  |  |  |
| ***Background and rationale*** | | |  |  |  |
| Provide a robust context and rationale for the proposed research in the *Introduction*. | Can discuss existing research, theory, and the wider context; the researcher is understood as *entering a conversation* with existing scholarship. | Critiquing the methodological limitations of existing research from a (post)positivist/ quantitative standpoint; orienting a literature review to finding a “gap” that the  research fills. | Introduction |  |  |
| Clearly articulate a research question – one that is methodologically coherent. | Can discuss refining an initially broader research question to a more specific one for the  paper. | Formulating research questions as hypotheses or expectations about what might be “found”. | Introduction |  |  |
| ***“Owning your perspectives”***^6^ | | |  |  |  |
| Include information on guiding theoretical assumptions and  other (e.g., explanatory) | Guiding (e.g., paradigmatic, ontological and  epistemological) and other | (Post)positivism and (simple) realism. | Study design |  |  |

| theory informing the use of TA. | theory should be coherent with RTA. |  |  |  |  |
| --- | --- | --- | --- | --- | --- |
| Report in a way that is consistent with stated theoretical assumptions throughout. | Theoretical coherence is evidenced through the use of language and concepts (e.g., around theme development, research subjectivity, data interpretation), the treatment  of data, and use of quality practices consistent with RTA. | Inadvertently “mashing-up” of RTA and (post)positivism/ realism (e.g., assuming data interpretation can be accurate and reliable) – without a clear rationale. | Study design  and Enhancing  Trustworthiness |  |  |
| Evidence methodological coherence/integrity in both the research and the report.^7^ | Theoretical assumptions, research questions, methods/practices of data generation, RTA, and specific orientation to RTA, purpose of research etc. all “fit together”,  conceptually. | Ontological and epistemological confusion (e.g., claiming constructionism but focusing on lived experience and treating language as a transparent window onto this). | Evident  throughout –  Introduction,  methodology  and conclusion |  |  |
| Show evidence of reflexive practice. | Can discuss researcher professional or personal positioning and experience in relation to the topic, and/or participant group, and/or their role in shaping the research;  use of reflexive journaling. | Evoking researcher bias (positivist), or even researcher influence, in a way that evokes it as *possible* rather than inevitable. | Dataset Generation  and Enhancing  trustworthiness |  |  |
| Write in a methodologically coherent style. | A first-person writing style suits RTA, as it “writes in” the researcher and contributes to situated and reflexive  reporting. | A third person writing style – writing the researcher out of the research. | First person  not used  due to  academic  writing style  for journals |  |  |
| **The Methodology**  NB: We prefer the theoretically-embedded term *Methodology* as a section header, over the proceduralist term *Method*. | | | Methodology |  |  |
| ***Participants/data items*** | | |  |  |  |
| Describe selection of participants/data items. | Should include criteria for selection and/or recruitment strategies and settings. | Terms “sample/sampling”, which connote “sampling” from a population (for the purpose  of statistical generalisation). | Participants  and context |  |  |
| Describe number of participants/data items; provide a rationale or explanation around dataset or participant group size/composition. | Non-positivist qualitative concepts, such as “information power” or sufficiency offer conceptually appropriate justifications for “dataset” or “participant group” size and composition.^8^ | Justification based on saturation (simple realist), or statistical models (positivist); reporting rates of non- participation (an indicator of the representativeness of the  “sample” in quantitative research). | Participants  and context |  |  |
| Discuss characteristics of participants/data items. | Balance the need to “situate the participant group” with participant anonymity (e.g., aggregate or report minimal demographics where  appropriate).^6^ | Tables with each participant’s demographic information listed line-by-line. | Participants  and context |  |  |

| Detail ethical approval and ethical code/principles followed, participant informed consent, etc. | Ethical discussion usually includes institutional ethical approval (if needed), but may include wider principles; providing research materials (participant information, consent form, etc.) in supplementary materials may be useful to support reflexive  openness. | Compromising participant anonymity by the details provided. | Ethical  Considerations |  |  |
| --- | --- | --- | --- | --- | --- |
| ***Dataset generation***  NB: We prefer the term *generation* over *collection* to capture the active role of the researcher and that data don’t pre-exist research *as* data, but *become* data through research practices. | | | Dataset  Generation |  |  |
| Provide some rationale for method(s) for data generation/data item sources chosen. | Discuss why the method(s) of data generation/data source was a good fit with the research question, participant group, guiding theory, etc. If multiple data sources are used, any rationale for combination should be conceptually appropriate (e.g.,  crystallisation^9^). | Triangulation as a rationale for different data sources (realist). | Dataset  Generation |  |  |
| Describe development and/or characteristics of data generation tool(s). | Include tool(s) in supplementary materials when possible; discuss piloting if used, and any changes following piloting, or during  data generation. | Using an existing tool with the aim of replicating existing  “findings”, or developing and describing a tool in a way that is intended to facilitate future  replication (positivist). | Dataset  Generation |  |  |
| Include details such as modality and/or setting of data generation, time frame, and other pertinent procedural information. | Relevant information includes: the mode of a data generation tool (e.g., video call focus groups; chat-based interviews); the context of data generation (location; timeframe) – where this  doesn’t compromise participant anonymity; and mode of recording interactive  data generation. | Standardisation as a gold standard (realist); justifying an aimed for standardisation in data generation tools as a means to facilitate the  “reliability” or “accuracy” of the research; treating a lack of standardisation in data generation method, modality or setting as a problem, a  potential source of “bias”. | Dataset  Generation |  |  |
| Describe who conducted any interactive data generation (which author or research role), and how. | Can include what, if anything, the researcher disclosed about their personal or professional positioning or motivation; what skills and experience they brought; note  researcher’s relationship with  participants prior to, during and after the research. | Seeking standardisation (e.g., through the training of researchers) in interactive data collection; treating non- standardisation as a threat to “reliability” or “accuracy”. | Dataset  Generation |  |  |
| Describe the size/scope of  dataset and dataset items. | Such as the range and average  length for interviews/focus | Equating data *quantity* with  data *quality.* | Dataset  Generation |  |  |

|  | groups; range and average word length for textual data items. |  |  |  |  |
| --- | --- | --- | --- | --- | --- |
| Describe, and if relevant explain, any preparation of data for analysis. | Such as method of transcription of audio/video data (a transcription key can go in supplementary materials); changes and “corrections” – such as why typographical errors in written data were corrected; system for removing any identifying information; use of pseudonyms and/or data  codes. | Describing transcription as “verbatim” or “orthographic” with no further details; using edited or “cleaned up” data without acknowledgement of this; participant validation of the “accuracy” of transcripts (realist). | Dataset  Generation |  |  |
| ***Data analysis*** | | |  |  |  |
| Provide some rationale for use of RTA, and, where relevant, for combining RTA with other approaches and procedures. | Any combining of RTA with other method/ologies or procedures should be warranted, rather than based on a misunderstanding of RTA, and conceptually coherent  (unless clearly justified). | Citing generic characteristics of RTA (e.g., accessible, flexible) without explaining how they were *relevant* to the study; using a codebook without acknowledging this is not part  of RTA and justifying its use. | Enhancing  trustworthiness |  |  |
| Describe specific orientation to RTA. | Locate RTA on dimensions of inductive<>deductive and semantic<>latent. | A generic discussion of TA (or even RTA), not specifically situated in relation to the study or approach. | Data analysis |  |  |
| Discuss how the researcher(s) engaged with the analytic process. | Provide a specific and situated account of the analysis process; use supplementary materials to provide a fuller  account of the analytic process. | Offering a generic description of the six phases of RTA in lieu of an account of analytic process. | Data analysis |  |  |
| Where more than one person is involved, describe who analysed the data (author or research role). | Role(s) or involvement throughout the process should be discussed; where coding was collaborative, what this involved and how differences in coding and theme  development were tackled, should be included. | Use of inter-coder agreement measures, consensus coding approach (positivist). | Data analysis |  |  |
| Use language to describe the process and products of RTA that is coherent with the values and assumptions of RTA. | Language should convey the *active* role of the researcher(s) in “generating”, “crafting”, “constructing”, “creating”, “producing” or “developing” themes; language around themes should evokes them as *products* of a researcher-data process. | Passive language of discovery, such as “emerging”, “found”, “identified”, “discovered” – these evoke themes as “diamonds scattered in the sand” (p. 740)^10^; unexplained use of language and concepts from other approaches, such as emergent or superordinate  themes (IPA), or line-by-line | Data analysis |  |  |

|  |  | and/or open coding and constant comparison (grounded theory). |  |  |  |
| --- | --- | --- | --- | --- | --- |
| **The Analysis**  NB: We prefer the heading *Analysis* over *Findings*/*Results*. *Findings* implies the researcher “found”, “discovered” or “identified” pre-existing themes. *Results* is strongly associated with the outputs of statistical analysis. | | | The  Analysis |  |  |
| ***Reporting the data analysis*** | | |  |  |  |
| Provide an overview of themes or thematic structure. | Overviews can include a list, map or table of themes to  preview the analysis. | An unclear thematic structure, including unexplained headings  in the *Analysis*. | The  Analysis  and  figure 1 |  |  |
| Ensure theme conceptualisation is appropriate to RTA, and any divergences are justified and explained. | In RTA, themes report shared meaning, united around a central organising concept that differs for each theme. | Topic summaries; data generation questions reported as “themes”. | The  Analysis  and  figure 1 |  |  |
| Name themes appropriately. | Use theme names that capture the “essence” or  “story” of each themes; brief  data quotations can be used. | (One-word) theme names that only identify a topic, and offer no story (evoking topic  summaries). | The  Analysis  and  figure 1 |  |  |
| Report themes in sufficient depth and detail. | As RTA is an interpretative method, themes should be multifaceted, and contain both data and analytic narrative; if useful, additional data extracts may be included  in supplementary materials.^7^ | Thin, one dimensional themes, effectively conflating codes and themes; large number of themes relative to the length of the manuscript. | The  Analysis and  Supplementary file  3 |  |  |
| Use subtheme judiciously. | Themes are the main analytic purpose, and should be multifaceted; only use subthemes where doing so highlights an important facet  or aspect of the central concept of a theme. | Fragmenting the analysis through overuse of subthemes, and an overly elaborated/“bitty” thematic structure. | The  Analysis |  |  |
| Ensure the analytic narrative explains the meaning and significance of the data. | For RTA, each theme needs an analytic narrative that outlines its meaning and importance in relation to the topic, research question and dataset; the reader needs to be told about why/how data excerpts matter and “evidence” the theme; the A*nalysis* section also needs to convey the *overall* story of the analysis. | Frequency counts as a justification for themes presented; simple paraphrasing of data as “analytic narrative”; treating data meaning as self- evident (data are assumed to speak for themselves); “arguing” with the data (treating the data as something to [dis]agree with, rather than to interpret and make sense  of). | The  Analysis |  |  |
| Provide an appropriate *balance* of analytic narrative and data extracts – both data  extracts *and* analytic narrative matter. | The rich descriptive and/or interpretative story of the analysis needs to be woven around sufficient analytic | Presenting either a long string of data extracts with barely any analytic narrative, or *only* the researcher’s narrative summary | The  Analysis |  |  |

|  | extracts from across the dataset. | of the theme, without any data extracts to support it. |  |  |  |
| --- | --- | --- | --- | --- | --- |
| Demonstrate coherence between analytic narrative  and illustrative/evidentiary data extracts. | Data extracts should convincingly and compellingly evidence the analytic claims. | Mismatches between data extracts and analytic claims;  not countering obvious alternative readings of the data | The  Analysis |  |  |
| Integrate existing research and theory into the analytic narrative. | In RTA, an interpretative analytic narrative is enriched by incorporating relevant existing research and theory into the reporting of themes, reflecting notions of contextualised meaning, and  contributing to an ongoing “conversation” about a topic. | The positivist tradition of separating a description of analytic “Results” and their interpretation with reference to scholarship and theory in a “Discussion” section. | The  Analysis |  |  |
| **The Final Section – A General Discussion or “Conclusions”**  NB: We don’t have a preference for what a final section of an RTA report is called, and it depends on the context and the focus and purpose of the study – the heading *Conclusion* may evoke a  certainty that isn’t appropriate; *Implications* may be useful; *Final Considerations* or *Reflections*  may work, as might *General Discussion*. | | |  |  |  |
| ***Quality, evaluation and conclusions*** | | |  |  |  |
| Draw analytic conclusions across themes. | Orient to the “so what” of the *overall* analysis – the “point” of the story told; this might include discussion of  implications for practice and “actionable” outcomes.^11^ | Repetitive theme-by-theme integration of the analysis with existing literature; no overall conclusions drawn; no overall analytic story. | Discussion |  |  |
| Discuss implications or directions for future research. | Any suggestions for future research should stem from the analysis and be evidence- based (e.g., provide grounds for other groups potentially  having different experiences or views) rather than generic. | Generic recommendations for other research, such as with a different “population”. | Implications for  Policy, practice  And further research |  |  |
| Use and report quality practices coherent with RTA. | Ensure evaluation of research quality deploys conceptually coherent notions, such as: member reflections; crystallisation;^9^ others serving as a critical friend/sounding  board to enhance insight;^12^ reflexive journaling. | Incoherent quality measures such as: member checking/participant validation; triangulation (realist); the use of theme agreement/consensus among researchers or  corroboration of themes by another researcher (positivist). | Enhancing  trustworthiness |  |  |
| Evaluate the research from a  *Big Q* standpoint. | Such evaluation might including considering how the *specifics* of the study may have shaped the research produced (for example, the characteristics and context of the participant group/dataset; the methods and modalities  for generating the data); | Evaluations and descriptions of limitations that orient to quantitative or positivist norms, such as reference to lack of generalisability – positioned as a limitation, and equated only with statistical generalisability^13^ – or a “small” | Strengths  and  Limitations |  |  |

|  | situatedness should not be treated as a limitation. | (by implication non-ideal) and “unrepresentative” “sample”. |  |  |  |
| --- | --- | --- | --- | --- | --- |
| Include reflections on | Some consideration of the | Reference to researcher |  |  |  |
| research process and | researcher(s)’s role in shaping | bias/influence (positivist). |  |  |  |
| practices, including researcher | the research and the |  |  |  |  |
| reflexivity. | knowledge generated is an |  | Enhancing  trustworthiness |  |  |
|  | important quality marker. |  |  |  |  |
